# Cutaneous Dermatologic Manifestations of Cardiovascular Diseases: A Narrative Review

**DOI:** 10.7759/cureus.72336

**Published:** 2024-10-24

**Authors:** Alan D Kaye, Rahib K Islam, Victoria T Tong, Elizabeth McKee, Julian J. Gonzales, Mohammed S. Rais, Abigail E. Watson, Christopher J Haas, Ryan Chan, Zachary Palowsky, Kazi N Islam, Sahar Shekoohi, Giustino Varrassi

**Affiliations:** 1 Department of Anesthesiology, Louisiana State University Health Sciences Center, Shreveport, USA; 2 School of Medicine, Louisiana State University Health Sciences Center, New Orleans, USA; 3 School of Medicine, University of Mississippi Medical Center, Jackson, USA; 4 School of Medicine, Florida State University College of Medicine, Tallahassee, USA; 5 Department of Dermatology, Louisiana State University Health Sciences Center, New Orleans, USA; 6 School of Medicine, New York Medical College, New York, USA; 7 Department of Agricultural Research Development Program, Central State University, Wilberforce, USA; 8 Department of Research, Fondazione Paolo Procacci, Rome, ITA

**Keywords:** atherosclerosis, cardiovascular disease, dermatology, heart failure, infective endocarditis, janeway lesions, skin, stasis dermatitis, takotsubo cardiomyopathy

## Abstract

Cardiovascular diseases represent the largest worldwide cause of morbidity and mortality. Common signs and symptoms of cardiovascular disease are well characterized and taught in medical curricula, allowing clinicians to quickly recognize and diagnose the more acute and emergent cardiovascular diseases. Dermatological features associated with cardiovascular diseases are less understood but very valuable to appreciate in clinical practice. The present investigation evaluates heart conditions such as heart failure, atherosclerosis, infective endocarditis, and Takotsubo cardiomyopathy, highlighting dermatologic signs that indicate underlying cardiovascular pathology and serve as important diagnostic and prognostic markers.

## Introduction and background

Cardiovascular diseases (CVDs) persistently remain among the largest contributors to global illness and mortality, impacting countless people across different populations [1]. These diseases encompass a wide range of both acute and chronic conditions, including but not limited to heart failure, atherosclerosis, and infective endocarditis, each with its distinct pathophysiological mechanisms and clinical presentations [2]. Traditionally, the diagnosis and management of CVDs have relied heavily on cardiovascular-focused assessments [3]. However, recognizing cutaneous manifestations associated with these diseases has gained increasing attention for its diagnostic and prognostic value [4]. As the body's largest organ, the skin can reflect internal systemic conditions, often serving as a window to underlying cardiovascular pathology [5]. Dermatologic signs may precede or coincide with the onset of cardiovascular symptoms, offering clinicians an early opportunity for diagnosis [6]. Furthermore, certain cutaneous findings can provide insight into the severity and progression of cardiovascular diseases, thus influencing patient management and outcomes [7]. The present investigation, therefore, examines the complex relationship between cardiovascular diseases and their related skin manifestations. By examining conditions such as heart failure, atherosclerosis, infective endocarditis, and Takotsubo cardiomyopathy, this review will highlight the dermatologic signs that indicate underlying cardiovascular pathology and serve as important diagnostic and prognostic markers. The aim is to highlight the importance of these skin manifestations in clinical practice, bringing them to clinicians’ awareness and promoting an interdisciplinary approach to managing patients with cardiovascular diseases. This review highlights underappreciated dermatologic manifestations of cardiovascular diseases, such as those associated with Takotsubo cardiomyopathy and cholesterol embolization syndrome, emphasizing their diagnostic and prognostic value. Unlike traditional cardiovascular evaluations, this paper advocates for integrating skin assessments into cardiovascular care, providing clinicians with early diagnostic clues and indicators of disease progression. By focusing on emerging and less recognized manifestations, the review offers a novel, interdisciplinary approach that enhances understanding of the skin-heart connection for improved patient outcomes. This review aims to explore the most commonly observed cutaneous manifestations in patients with cardiovascular diseases and investigate how these dermatologic findings can aid in early diagnosis and prognosis.

## Review

Methods

In conducting this narrative review, a comprehensive literature search was performed using medical databases such as PubMed and Embase to identify relevant studies on cutaneous manifestations associated with cardiovascular diseases. The search terms included combinations of keywords such as “cutaneous manifestations,” “dermatology,” “cardiovascular diseases,” “prognostic value,” and “early diagnosis.” Studies published between 2000 and 2024 in peer-reviewed journals were considered for inclusion. The review focused on research involving adult patients (aged 18 and above) with cardiovascular conditions such as heart failure, atherosclerosis, infective endocarditis, and Takotsubo cardiomyopathy. Articles were included if they reported on dermatologic signs and symptoms observed in patients with cardiovascular diseases. Exclusion criteria comprised studies exclusively focused on pediatric populations, non-English language articles, and case reports with insufficient clinical data or lacking relevance to the research question.

Heart failure

Heart failure (HF) arises due to abnormal function of valves or ventricles from a variety of preceding conditions, including hypertension, resulting in characteristic signs and symptoms. The heart’s ability to fill and eject blood can be negatively impacted by improper function. As a result of this aberrant blood flow, one sign of HF is elevated central venous pressure. Well-known risk factors for developing HF include but are not limited to ischemic heart disease, hypertension, cardiomyopathies, substance abuse such as with alcohol and cocaine, and autoimmune diseases [8]. With such a high prevalence of these risk factors and HF itself, it is imperative to understand the relationship between HF and its cutaneous manifestations. One such manifestation often linked with HF is stasis dermatitis.

Cutaneous Manifestations

Stasis dermatitis represents a subtype of inflammatory dermatitis, primarily seen in elderly patients with venous insufficiency of the lower extremities secondary to inadequate valve function. Due to the elevated venous pressure with stasis dermatitis, erythrocytes, macrophages, plasma, electrolytes, leukocytes, and fibrin extravasate induce matrix metalloproteinase (MMP) secretion. With increased MMPs in the extracellular matrix, the resultant remodeling manifests as venous ulcers and lesions of the lower extremities, characteristic of the diagnosis [9]. While stasis dermatitis is most commonly associated with chronic venous insufficiency, it is essential to recognize that patients with heart failure can exhibit similar lower extremity skin changes due to elevated central venous pressure. In these cases, the resulting pitting edema and erythema may mimic the dermatologic manifestations of chronic venous insufficiency. In heart failure, chronic venous hypertension and impaired lymphatic drainage lead to lower extremity edema, which predisposes the skin to stasis dermatitis. This condition often presents as erythema, scaling, pruritus, and hyperpigmentation of the lower legs. Lipodermatosclerosis (skin thickening due to the onset of fibrosis) may occur further along and in advanced cases. These changes make the skin vulnerable to trauma, resulting in venous ulcers. Chronic heart failure patients with stasis dermatitis may develop venous ulcers. These are usually shallow, irregularly shaped wounds located above the medial malleolus. They can become secondarily infected, further complicating the management [9]. Lastly, another dermatological manifestation of chronic heart failure can lead to poor peripheral perfusion, resulting in a pallor or cyanotic appearance of the skin, especially in the extremities. This occurs due to decreased oxygen delivery and venous stasis, often worsening with decompensation [4,8]. However, when these skin findings appear in the context of heart failure, they should be understood as a consequence of impaired cardiac function rather than primary venous pathology [4]. In clinical practice, it is essential to recognize the relationship between HF and stasis dermatitis and their diagnostic and prognostic significance. Another common and clinically relevant cutaneous manifestation of heart failure is leg edema [5]. Edema in heart failure typically presents as bilateral pitting edema, which occurs due to increased central venous pressure and subsequent fluid retention in the lower extremities. However, it is essential to differentiate this from other types of edema based on their etiology. For instance, lymphedema is characterized by non-pitting swelling, which may be unilateral or bilateral and can be seen on the skin as a form of thickening or fibrosis, referred to as peau d'orange. In contrast, venous stasis edema, commonly in chronic venous insufficiency, is pitting and often associated with skin changes like hyperpigmentation, lipodermatosclerosis, and ulcerations [9]. Recognizing these distinctions is crucial for clinicians as it aids in identifying the underlying cause and guiding appropriate treatment strategies.

Diagnostic and Prognostic Significance

Kaya et al. conducted an observational cross-sectional study with an enrollment of 158 patients admitted to an HF outpatient clinic. Before this study, there was a lack of research on the relationship between stasis dermatitis and HF outcomes. The study showed that the presence of stasis dermatitis and coronary artery disease was independently associated with the risk of HF-related hospitalization. Therefore, for the first time, stasis dermatitis was determined to be an independent predictor for HF-related hospitalization [10].

With length of stay (LOS) being the primary driver of hospitalization costs in heart failure patients, Kaya et al. investigated the relationship between stasis dermatitis and LOS in HF patients. The mean LOS was 5.4 ± 2 days, while “prolonged LOS” was defined as LOS >5 days. Their study was the first to show that stasis dermatitis was associated with the risk of more extended hospitalization independent of other factors in patients with heart failure with reduced ejection fraction (HFrEF) admitted for acute decompensated HF [11].

Atherosclerosis

Atherosclerosis is a chronic, systemic inflammatory disease impacting the vasculature and is a significant contributor to the leading cause of death globally. While this condition has various contributing factors, it is believed to be shaped by a blend of genetic and environmental influences, including hypertension, diabetes, smoking, and elevated cholesterol levels. These risk factors contribute to the molecular mechanisms of the underlying pathophysiology, resulting in the disruption of arterial wall cells and putting individuals at risk for myocardial infarctions and stroke [12]. Accumulated low-density lipoprotein (LDL) proteins initiate a pro-inflammatory state, leading to the upregulation of adhesion molecules on the endothelial surface and the recruitment of monocytes and macrophages. Macrophages consume the excess LDL proteins, forming foam cells, which form cholesterol crystals that release IL-1B and IL-6, further exacerbating the proinflammatory effects. Apoptosis of smooth muscle cells and macrophages can result as the lesion advances, accumulating debris that forms the necrotic, lipid-rich core of the atheroma [13]. This plaque of the arterial intima disrupts the endothelial layer and activates the coagulation cascade, increasing the risk of occlusion of arterial vessels [13].

Cutaneous Manifestations

The skin is the second most common organ system affected by cholesterol embolization syndrome (CES). Shah et al. reported that the kidneys are the most commonly affected organ system (31.5%), followed by the skin (15.5%) and gastrointestinal (GI) tract, all of which can present with organ-specific manifestations. The "blue toe syndrome" is a hallmark of CES, presenting as a sudden onset of blue or purplish discoloration of the toes, usually accompanied by pain due to tissue ischemia. Microembolization may also result in ulcerations and gangrene if not promptly addressed [14]. Skin involvement results in lesions such as livedo reticularis, a finding of red-blue, net-like, and lacey patches that result from decreased blood flow to the skin and microvascular ischemia. A common skin finding associated with atherosclerosis is characterized by a netted, almost mottled-like pattern of red-blue skin discoloration. It is caused by occlusion or reduced flow within the cutaneous vasculature, reflecting microvascular ischemia [15]. Other characteristic findings include retiform purpura, blue or purple toes, cyanosis, small nail bed infarcts, ulceration, and gangrene if blood flow is not restored. Lesions most commonly affect the skin of the lower extremities, with asymmetric findings if both extremities are involved [15]. Other diagnostic clues include microembolism-induced symptoms, such as transient visual disturbances or amaurosis fugax, which may indicate retinal emboli. Tuberous or tendinous xanthomas may form in severe states of hypercholesterolemia associated with atherosclerosis. These are solid, firm-like, yellowish nodules often found on the outside-extensor surfaces of the limbs or over the Achilles tendons. Additionally, patients may present with systemic symptoms like fever, myalgia, and weight loss due to an inflammatory response triggered by embolization. Laboratory findings can also support the diagnosis; for instance, elevated eosinophil counts (eosinophilia) and increased serum creatinine levels may accompany skin manifestations, reflecting organ involvement. Biopsy of affected skin lesions, such as retiform purpura or livedo reticularis, can reveal cholesterol clefts within arterioles, further confirming the diagnosis [14,15].

Cardiovascular Manifestation

Cholesterol emboli syndrome is closely related to atherosclerosis due to the pathogenesis of emboli formation from atherosclerotic plaques. If cholesterol crystals break off from this plaque, end-organ damage can result from the mechanical obstruction of vascular beds and inflammatory response to the target organ [14]. Embolization is mostly iatrogenic or induced by interventional and surgical cardiovascular procedures but can also occur spontaneously [14,16]. Complications of cholesterol embolization depend on which organ system is involved, with major cardiac complications including hypertension, carotid stenosis, end-organ ischemia, and myocardial ischemia [14]. Many patients who suffer from a cholesterol embolus have underlying atherosclerotic heart disease and have atheromatous plaques in large caliber arteries such as the aorta and its branches [14].

Diagnostic and Prognostic Significance

The risk of cholesterol emboli syndrome is directly related to the severity of concurrent atherosclerosis [14,16]. Certain atherosclerotic plaque characteristics increase the risk of cholesterol emboli syndrome, including plaque ulceration, thickness ≥ 4 mm, and mobile thrombi. Transesophageal echocardiogram, computed tomography, and magnetic resonance imaging can be useful in evaluating atherosclerotic plaques of high-risk individuals with risk of CES [16]. Ozkok, 2019, points out that CES is a manifestation of advanced atherosclerosis, so one can prevent further advancement of cardiovascular disease by addressing underlying causes of atherosclerosis and controlling systemic inflammation [16]. Non-pharmacologic measures can be used for secondary prevention, including blood pressure control, glycemic control, weight management, smoking cessation, and medications such as aspirin, statins, ACE inhibitors (Angiotensin-Converting Enzyme inhibitors), and Angiotensin Receptor Blockers (ARBs)** **[16].

Infective endocarditis

Infective endocarditis (IE) is inflammation of the endocardial lining of the cardiac valves and chambers resulting from a bacterial or fungal infection [17]. IE is categorized into acute IE, which begins suddenly and progresses rapidly over a few days, or subacute endocarditis, which develops gradually and may persist for weeks to months [18]. Acute bacterial endocarditis is commonly caused by gram-positive *Staphylococcus*, *Streptococci*, and *Enterococci* [17]. Subacute bacterial endocarditis is most commonly caused by *Streptococcus viridans* and other bacteria found in the oropharynx [19]. Fungal endocarditis is a rare condition that may be caused by *Candida*, *Histoplasma*, and *Aspergillus* species [20]. IE presents with cardiovascular manifestations that progress to extracardiac complications if not diagnosed and treated in a timely manner [17]. 

Cardiovascular Manifestations

The bacteremia associated with IE results in vegetation on heart valves composed of fibrin, platelets, and infecting organisms held together by agglutinating antibodies [21]. Vegetations cause heart valve dysfunction and damage that can be heard as new or changing murmurs during auscultation in 75-85% of patients [22]. Damage to the heart and its chambers can result in arrhythmia and, eventually, congestive heart failure. Over 80% of IE patients with aortic valvular insufficiency and 50% of IE patients with mitral valvular insufficiency developed heart failure [23]-septic emboli form when the vegetations dislodge into the bloodstream and obstruct a blood vessel [24]. Septic emboli occur in various organ systems and cause tissue damage, resulting in extracardiac manifestations and even death associated with IE [25]. Septic emboli associated with IE were most commonly found in the brain, kidneys, spleen, and lungs [26].

Cutaneous Manifestations

Patients with IE have dermatological manifestations up to 20%, ranging from non-specific petechiae to more specific skin lesions, such as Janeway and Osler nodes [27]. Petechiae are small pinpoint (< 2 mm), non-blanching mucocutaneous lesions seen in 20-40% of IE patients [28,29]. Subungual splinter hemorrhages are a rare manifestation of subacute endocarditis that results in non-blanching vertical lines of hemorrhage seen underneath the nailbed [30]. Petechiae and splinter hemorrhages are associated with numerous disease manifestations and may arise idiopathically. Although splinter hemorrhages only have a sensitivity of 26% (95% CI 22 to 31) in clinically suspected cases of IE, they still have a specificity of 83% (95% CI 79 to 86), indicating that they are of clinical significance in the diagnosis of IE [31]. Dermatologically, they can be described as small, pinpoint hemorrhagic spots and are the most common dermatological manifestation of infective endocarditis. They typically appear on mucosal surfaces (e.g., conjunctiva, oral mucosa) or extremities and may signal systemic embolization [27,30,31].

Janeway lesions and Osler’s nodes are forms of palpable purpura highly suggestive of IE [32,33]. Janeway lesions are found in less than 10% of all cases of IE, whereas Osler’s nodes may be seen in up to 10-25% of all cases [34]. It was initially believed that Janeway lesions were a vascular phenomenon, whereas Osler’s nodes were an immunologic phenomenon of IE [35]. Histological examination of both Janeway lesions and Osler’s nodes reveals micro-emboli and micro-abscesses; however, biopsies taken later in the course of Osler’s nodes may show immunologic phenomena [35]. Although once believed to have distinct pathogenesis, it is generally accepted that Janeway lesions and Osler nodes result from septic micro-emboli forming micro-abscesses in the dermis.33 Janeway lesions are small, painless, erythematous, or hemorrhagic lesions on the palms and soles [36,37]. Janeway lesions are associated with acute IE and may last days to weeks before completely healing [38]. Unlike Janeway lesions, the development of Osler’s nodes is associated with pain [35]. Osler’s nodes are painful, raised, red-purple nodules found on the fingers and toes [36]. Osler’s nodes are associated with subacute IE and may last hours to days [38]. Dermatologically, these pathological findings can be described as non-tender, erythematous, or hemorrhagic macules that appear upon an individual's palms and soles. Their painless nature differentiates them from Osler's nodes. Histologically, they show microabscesses with neutrophil infiltration linked to septic embolization [33,34]. On the other hand, Osler's nodes can dermatologically be described as erythematous nodules located on the fingertips or toes [38]. Lastly, splinter hemorrhages can dermatologically be described as non-blanching, linear, reddish-brown streaks seen beneath the nails [30].

Diagnostic and Prognostic Significance

According to the modified Duke criteria, cutaneous manifestations are minor supporting criteria for diagnosing IE [39]. IE presents with many nonspecific symptoms, and most patients do not have any clinically significant symptoms until after embolism occurs [40]. Cutaneous manifestations in IE patients are associated with the dissemination of infectious and inflammatory products into the systemic vasculature; therefore, cutaneous signs are also linked to increased rates of complications and worsening prognosis in these patients [32,41]. A study performed in 2014 found that IE patients with purpura had larger cardiac vegetation (P=0.01), and the presence of Janeway lesions was associated with increased extracerebral emboli (P=0.02) [42]. Screening for cutaneous manifestations of IE and early clinical intervention may significantly reduce disease morbidity and mortality [35].

Takotsubo cardiomyopathy

Takotsubo cardiomyopathy (TC), often referred to as stress cardiomyopathy, is a temporary and reversible condition characterized by a unique apical ballooning appearance of the left ventricle [43]. It primarily affects postmenopausal women and is typically triggered by emotional or physical stress, with a notable absence of significant coronary artery disease [44]. The hallmark of TC is transient left ventricular systolic dysfunction, which is believed to result from an excess release of catecholamines, leading to catecholamine-induced cardiotoxicity and microvascular dysfunction [45]. These mechanisms have been hypothesized to be intertwined with complex neuroendocrine processes that involve the cognitive centers of the brain as well as the hypothalamic-pituitary-adrenal axis. Excessive catecholamines released by the sympathetic nervous system during stress may cause intracellular calcium overload in myocardial cells, mediated through β1-adrenoreceptor signaling pathways [46]. This calcium overload can result in ventricular dysfunction, contributing to the characteristic cardiotoxicity seen in TC [45]. Common symptoms include chest pain and dyspnea, although more severe manifestations, such as serious ventricular arrhythmias and cardiac arrest, may also occur [47]. Despite its clear clinical presentation, TC remains a diagnosis of exclusion, necessitating thorough evaluation to rule out other conditions, such as acute myocardial infarction [43]. The characteristic catecholamine surge leads to elevated plasma levels of epinephrine, significantly higher than those observed in myocardial infarction (MI) patients, particularly under stress [43].

Cutaneous Manifestations

Acral ischemia syndrome is characterized by diminished blood flow to the extremities, particularly affecting the fingers, toes, ears, and nose. It is frequently associated with systemic conditions such as vasculitis, autoimmune disorders, and malignancies [48]. The pathophysiology of acral ischemia syndrome is not fully understood and is likely multifactorial. Potential mechanisms include vaso-occlusion of vessels due to vasoconstrictive factors produced by malignant cells [49]. Additionally, the compressive effect of tumors on the cervical plexus may lead to hyperstimulation of the sympathetic nervous system, resulting in vasospasm. Other proposed mechanisms include immune complex deposition, micro-embolization of tumor cell fragments, increased coagulability due to elevated circulating procoagulant factors, spontaneous platelet aggregation, or impairment of fibrinolytic and anticoagulant pathways [50]. In the initial stages, patients might exhibit cold, pale, or bluish extremities, frequently accompanied by sensations of numbness, tingling, or burning in the affected areas. As ischemia progresses, it can result in more severe symptoms, including pain, the formation of ulcers, or even gangrene [48].

Cardiovascular manifestations and diagnostic and prognostic significance

The relationship between TC and acral ischemia syndrome is not well-established in the current literature, yet emerging evidence suggests that these two conditions may share common underlying mechanisms. Stress or emotional response can precipitate both due to heightened sympathetic nervous system activity, leading to vasoconstriction and transient left ventricular dysfunction [51]. Both TC and acral ischemia syndrome may have shared risk factors, including chronic stress, underlying autoimmune disorders, or pre-existing vasculopathy. Another potential shared mechanism is microvascular dysfunction through impaired endothelial function, causing failure to regulate blood flow [48,51].

Diagnostic implications

Importance of Dermatologic Signs in the Early Diagnosis of Cardiovascular Diseases

Data reported by the World Health Organization and the American Heart Association report that cardiovascular pathologies are the leading causes of untimely and early death [52]. Research indicates that distinct dermatologic signs observed during routine examinations can play a vital role in the early detection and diagnosis of cardiovascular diseases. Various skin manifestations such as xanthomas, acanthosis nigricans, and specific rashes can signal underlying cardiovascular issues like hyperlipidemia, diabetes mellitus, and infective endocarditis. Krishnan et al. reported a case of an 11-year-old female presenting with ichthyosis vulgaris [53]. Upon further testing, she was diagnosed with dilated cardiomyopathy with severe biventricular dysfunction. The keen observations of ichthyosis vulgaris in conjunction with other signs such as pedal edema, shortness of breath on exertion, and abdominal pain helped her clinicians arrive at the gestalt diagnosis. This case report highlights the importance of recognizing dermatological signs, such as the skin and the heart. However, separate organs are highly linked together physiologically, as when the heart fails, the skin shows [3]. Other inflammatory conditions such as psoriasis, hidradenitis suppurative, and systemic lupus erythematosus (SLE) are also linked with cardiovascular disease [52]. These conditions are more readily identifiable manifestations due to their prevalence. Not so easily seen is the buildup of inflammatory markers, such as various interleukins, which can lead to endovascular plaque formations, contributing to cardiovascular pathology [52]. Dermatologists and other medical doctors can recognize these signs, leading to timely cardiovascular workups. As such, necessary intervention, improving patient outcomes, ​ and reducing patient mortality become more feasible with rapid identification and intervention.

Prognostic implications

How Cutaneous Manifestations Can Indicate Disease Progression and Prognosis

Cutaneous manifestations can provide valuable prognostic information about cardiovascular diseases in susceptible patients. Heart pathologies manifest externally via sentinel cutaneous findings, and the type and degree of dermatologic pathology can serve as a vital indicator for clinicians to recognize disease progression and gauge prognosis more adequately.

For instance, in patients with peripheral vascular disease, severe chronic venous insufficiency often presents with skin changes, ranging from varicose veins due to faulty valves to lipodermatosclerosis and venous ulcers. These cutaneous manifestations indicate advanced disease and a higher risk of cardiac and vascular complications. Similarly, livedo reticularis and necrosis can be associated with systemic vasculitis and severe atherosclerosis, suggesting a poorer prognosis [53].

Another common finding of atherosclerosis and peripheral vascular disease is non-healing ulcers, whether they are associated with diabetes and neuropathy or atherosclerotic arterial disease. Patients with diabetic ulcers, most commonly of the foot, have significant risk factors for heart failure. All clinicians need to recognize these manifestations to appropriately direct patients to the care they require [54], also thinking of the frequently associated neuropathy [55]. Moreover, they should not forget that appropriate nutrition may be beneficial, both for atherosclerosis and for neuropathic pain (Table 1) [56].

**Table 1:** Cutaneous Manifestations of Cardiovascular Diseases HF: heart failure; CES: Cholesterol embolization syndrome

Cardiovascular Disease	Cutaneous Manifestation	Prevalence/Incidence	Clinical Features	Diagnostic Significance	Prevention/Treatment
Heart Failure	Stasis dermatitis	Common in chronic cases [25].	Erythema, scaling, pruritus, hyperpigmentation in lower legs	Indicates venous hypertension and poor cardiac function	Compression therapy, leg elevation, emollients
	Venous ulcers	Frequent in advanced HF [25].	Shallow ulcers above medial malleolus, irregular shape	Sign of severe venous insufficiency, risk for infection	Wound care, compression, antibiotics if infected
Atherosclerosis	Livedo reticularis	~15.5% in CES cases [13].	Mottled, net-like reddish-blue pattern on skin	Reflects microvascular ischemia due to cholesterol embolization	Managing cholesterol levels, antiplatelet therapy
	Blue toe syndrome	Associated with CES [16].	Blue discoloration of toes, painful	Indicates cholesterol microemboli; can precede organ ischemia	Revascularization, anticoagulation
Infective Endocarditis	Janeway lesions	<10% [19].	Painless, erythematous macules on palms and soles	Suggestive of septic emboli, usually seen in acute IE	Antibiotic therapy
	Osler's nodes	10-25% [19].	Tender, red-purple nodules on fingertips or toes	Associated with subacute IE, indicates immune complex deposition	Antibiotic therapy
Takotsubo Cardiomyopathy	Acral ischemia	Less common, case reports [45].	Cold, pale, or cyanotic extremities; may progress to ulceration	May indicate systemic vasospasm or severe microvascular dysfunction	Vasodilators, treatment of underlying stress triggers
	Raynaud’s phenomenon	Rare, linked to stress [48].	Transient blanching and cyanosis of the digits	Reflects vasospasm, can be triggered by emotional or physical stress	Avoiding triggers, calcium channel blockers

Summary of key studies

A summary of clinical studies has been mentioned in Table 2.

**Table 2:** Summary of Key Studies Examining Cutaneous Manifestations in Cardiovascular Diseases

Author(s) and Year	Study Design	Key Findings	Cutaneous Manifestations Examined	Diagnostic/Prognostic Significance
Kaya & Kaya, 2024 [10].	Observational Cross-sectional Study	Stasis dermatitis was identified as an independent risk factor for heart failure (HF)-related hospitalization. Significant associations were found with diabetes mellitus, Chronic Obstructive Pulmonary Disease (COPD), and increased pulmonary artery pressure.	Stasis Dermatitis	Diagnosis of stasis dermatitis indicated poor prognosis and increased risk of HF-related hospitalization.
Ozkok, 2019 [16].	Review of Current Perspectives	Cholesterol-embolization syndrome (CES) is a multisystemic disease caused by embolization of cholesterol crystals (CCs). Embolized CCs lead to ischemic and inflammatory damage, and anti-inflammatory agents have been explored as potential treatments.	Livedo Reticularis, Blue Toe Syndrome	CES manifests with livedo reticularis and blue toe syndrome, indicating systemic cholesterol embolization from atherosclerotic plaques.
Servy et al., 2014 [42].	Observational, Prospective Epidemiological Study	Found that 11.9% of infective endocarditis (IE) cases presented with skin manifestations such as Osler’s nodes, Janeway lesions, and purpura. Patients with skin manifestations had higher rates of extracardiac complications.	Osler’s Nodes, Janeway Lesions, Purpura	Skin manifestations in IE patients were linked to larger cardiac vegetations and a higher rate of extracerebral emboli, indicating poor prognosis.
Krishnan et al., 2022 [53].	Case Report	Reported a case of an 11-year-old with ichthyosis and dilated cardiomyopathy, successfully managed with heart transplantation. Highlighted the role of cutaneous manifestations as markers of life-threatening cardiac conditions.	Ichthyosis	Ichthyosis in the patient was linked to dilated cardiomyopathy, underscoring the importance of recognizing skin manifestations that could indicate severe cardiac disease.
Topan et al., 2015 [39].	Prospective Observational Study	The study evaluated the diagnostic accuracy of the modified Duke criteria for infective endocarditis (IE). Among 241 patients, 137 were classified as having definite IE, and 43% of cases would become possible IE upon removal of major microbiological criteria.	Peripheral Vascular Stigmata (Osler's Nodes, Janeway Lesions, Splinter Hemorrhages)	Peripheral vascular stigmata were highlighted as key diagnostic features within the Duke criteria, aiding in the diagnosis of definite IE.

Clinical practice implications

Recommendations for Clinicians on Integrating Dermatologic Assessments in Cardiovascular Disease Management

Physicians of all specialties are encouraged to utilize their physical exam and observation to better care for patients with underlying cardiac pathologies and to detect cutaneous manifestations linked to underlying diseases [6]. Given their chief complaint and family history, physicians are advised to incorporate thorough dermatologic assessments into the routine evaluation and workup of patients with or at risk for cardiovascular diseases. Upon recognizing various cutaneous manifestations, physicians should consult dermatology and cardiology and refer the patient to an appropriate specialist for an appointment to receive more specialized care based on the patient’s chief complaint, family history, and current presentation. Recognizing and interpreting cutaneous signs can prompt early diagnostic testing and timely management of cardiovascular conditions [6]. Dermatologists should collaborate with cardiologists to ensure comprehensive care, particularly in patients presenting with unexplained skin findings that can suggest cardiovascular pathology.

**Figure 1:**
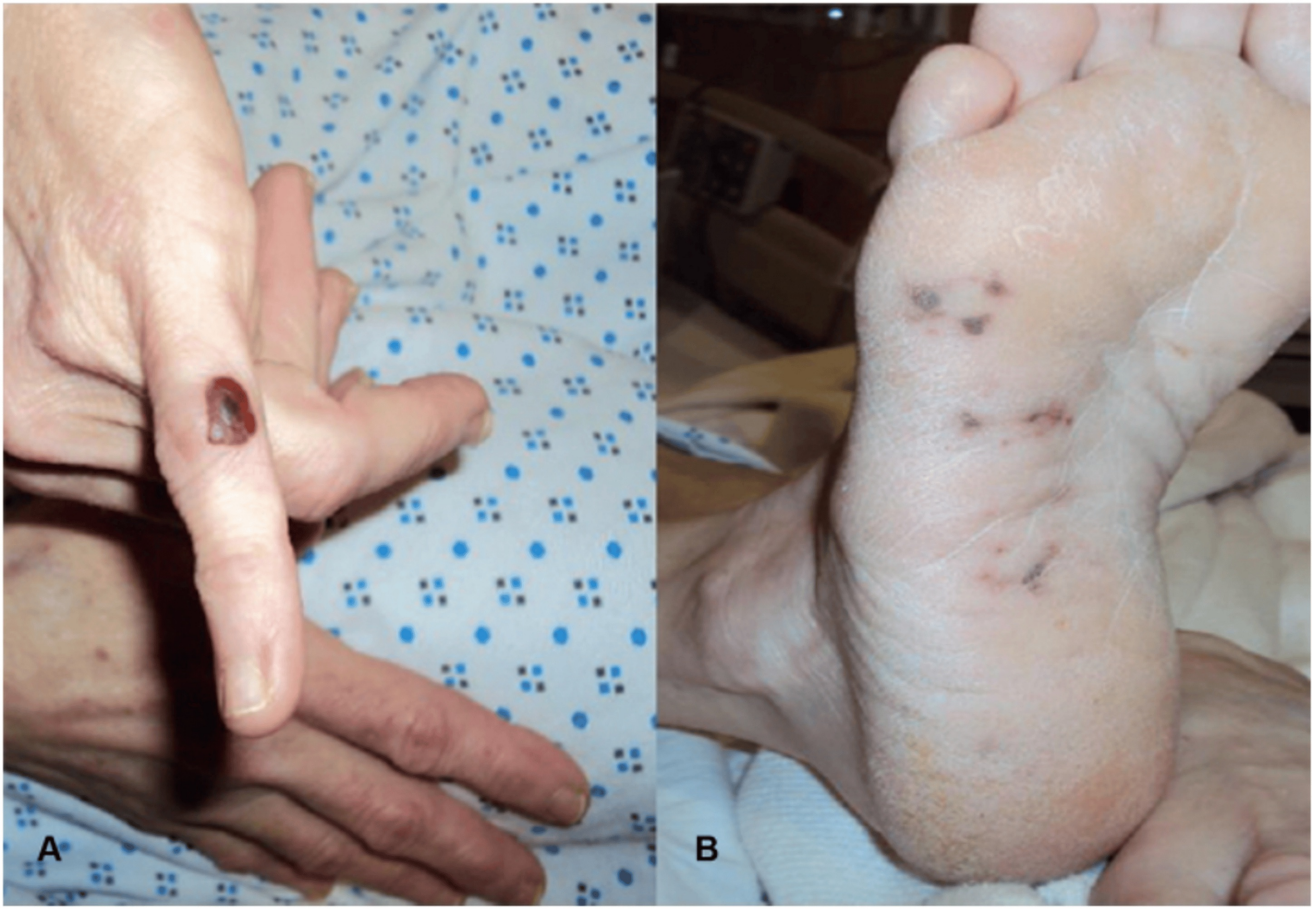
Cutaneous Manifestations of Infective Endocarditis. (A) Osler’s Node (B) Janeway lesions Source: Rochlani et al. [57]. Available for use under Open Access License - Creative Commons Attribution – NonCommercial – NoDerivs (CC BY-NC-ND 4.0) (https://creativecommons.org/licenses/by-nc-nd/4.0/)

The following section will present visual representations of the vital cutaneous manifestations associated with cardiovascular diseases. These images enhance the understanding of the clinical features described earlier, offering readers a more comprehensive view of how these dermatologic signs manifest in real-world cases. By examining conditions such as stasis dermatitis, livedo reticularis, blue toe syndrome, and others, these images will underscore the diagnostic and prognostic significance of recognizing such cutaneous manifestations. Each figure is annotated to highlight the defining characteristics, providing clinicians and researchers with a valuable reference for comparison in practice (Figure 1) [57].

## Conclusions

In summary, the interplay between cardiovascular diseases and their cutaneous manifestations is a crucial yet often underappreciated aspect of clinical medicine. This review has explored the association between several major cardiovascular conditions: failure, atherosclerosis, infective endocarditis, and Takotsubo cardiomyopathy and their corresponding dermatologic signs, such as stasis dermatitis, cholesterol embolization syndrome, Janeway lesions, and acral ischemia syndrome. These manifestations not only provide valuable diagnostic clues but also serve as important prognostic markers, offering insights into the severity and progression of the underlying cardiovascular disease. Despite the established connections between cardiovascular diseases and their cutaneous manifestations, there remain significant gaps in our understanding. Future research should clarify the pathophysiological mechanisms that drive these associations and consider the potential for systematically incorporating dermatologic assessments into cardiovascular care. Encouraging closer collaboration between dermatologists and cardiologists can enable healthcare providers to adopt a more holistic approach to patient care, ultimately improving clinical outcomes. As we continue to deepen our understanding of the skin-heart connection, clinicians must remain vigilant in recognizing the cutaneous manifestations of cardiovascular diseases, thereby optimizing patient management and care.
